# Energy Harvesters and Self-Powered Sensors for Smart Electronics, 2nd Edition

**DOI:** 10.3390/mi15010099

**Published:** 2024-01-04

**Authors:** Qiongfeng Shi, Huicong Liu

**Affiliations:** 1 Interdisciplinary Research Center, School of Electronic Science and Engineering, Southeast University, Nanjing 211189, China; 2 School of Mechanical and Electrical Engineering, Jiangsu Provincial Key Laboratory of Advanced Robotics, Soochow University, Suzhou 215123, China

## 1. Introduction

With the worldwide rollout of the 5G communication network and 6G around the corner, we have witnessed the rapid development of the Internet of Things (IoT) technology, enabling big data and digital transformation in various fields [1], e.g., remote healthcare, intelligent body area network, smart farming, smart building, industry 4.0 and smart transportation. The successful implementation and functionality of IoT networks require support from an enormous number of interconnected sensor nodes for localized and holistic information acquisition. The conventional power supplying strategy using batteries possesses inevitable drawbacks of a limited lifespan, bulky/heavy characteristics, environmental unfriendliness and difficulty in replacement, greatly hindering the sustainable development of IoT networks. In this regard, energy harvesting technologies that can scavenge available energy (e.g., light, thermal, kinetic and radio frequency energy) from the surroundings appear to be promising candidates in combination with batteries for sustainable power supply in the IoT era [2]. The widely adopted energy harvesting technologies include piezoelectric, triboelectric and electromagnetic mechanisms for kinetic energy [3,4,5]; thermoelectric and pyroelectric mechanisms for thermal energy [6,7]; photovoltaic effect for light energy [8]; and antenna rectification for radio waves [9]. In practical implementation, energy harvesting devices can be configured into energy harvesters for continuous power feeding or self-powered sensors for zero-powered sensing [10], both contributing to higher sustainability in standalone IoT sensor nodes. The research field is currently focused on achieving output performance improvement, efficient power management and compact system integration via mechanism, material, structure and circuit innovation, paving the way toward functional and self-sustainable IoT nodes and systems.

This Special Issue is the second edition of “Energy Harvesters and Self-Powered Sensors for Smart Electronics”, and it showcases the continuous and important progress in the field of energy harvesting and self-powered sensing. This edition contains 10 published articles (9 research articles and 1 review article) that address the urgent demand of sustainable power supply for IoT systems, aiming to further advance the field through detailed explorations of device design, circuit optimization, strategy enhancement and system integration.

## 2. Overview of the Published Articles

Our ambient surroundings contain abundant mechanical energy sources that are not fully used in most circumstances, including vibrations, rotation, wind, water waves, raindrops and human motions. To efficiently harvest the mechanical energy in low-frequency ranges, Li et al. (contribution 1) proposed a piezoelectric rotational energy harvester with a dual-frequency up-conversion design. The energy harvester consists of multiple piezoelectric beams that undergo high-frequency self-excitations for efficient power generation under the trigger of a multi-paddle slider. The testing results indicate that the designed rotational energy harvester with three piezoelectric beams can produce an output power and a power density of 5.392 mW and 4.02 μW/(cm^3^ Hz), respectively, when driven by a 0.42 Hz rotational excitation. Meanwhile, Zhang et al. (contribution 2) presented a push–pull diamagnetic levitation design for airflow energy harvesting using the electromagnetic mechanism. To compensate for the rotor offset and ensure higher efficiency, the floating rotor is configured with a symmetrical four-notch structure. The energy harvester is able to generate a peak voltage of 2.709 V (40.80% improvement over a three-notch structure) when subjected to a 3000 sccm airflow. The corresponding output power is 138.47 mW, showing great potential in scavenging the ambient airflow energy.

Radio frequency (RF) electromagnetic waves are another common form of renewable energy in the environment due to the widely deployed wireless communication networks. Li et al. (contribution 3) proposed a fully on-chip solution for tunable voltage boosting (TVB) to improve the acquisition performance of low-power RF signals. With the optimal TVB design, the output voltage of the rectifier can be maintained at a high level of 1 V across the new broad 5G radio frequency bandwidth from 3 to 6 GHz. Typically, an output voltage and a peak power conversion of 1 V DC and 83% are achieved at 3 GHz when an input power of −23 dBm is applied. Concurrently, the same group (contribution 4) further developed a low-dropout regulator (LDO) across multi-voltage domains using the 180 nm complementary metal oxide semiconductor (CMOS) technique for RF energy harvesting. The as-fabricated chip exhibits a small area of only 0.149 mm^2^, although it is able to deliver a large current of up to 400 mA. For the low-voltage domain, the LDO has a line regulation of 1.85 mV/V and a load regulation of 0.0003 mV/mA. However, for the high-voltage domain, these parameters increase to 3.53 mV/V and 0.079 mV/mA. The small form factor and the low standby power consumption (174.5 µW) make the LDO an ideal candidate for powering IoT nodes using an RF energy harvesting chip.

To enable self-sustained IoT remote traffic sensing, Dipon et al. (contribution 5) presented a hybrid energy harvester that can simultaneously scavenge the mechanical vibration from passing vehicles and the temperature gradient from the asphalt and the soil underneath the road. The hybrid device consists of a stacked piezoelectric generator and a thermoelectric generator with respective AC-DC and DC-DC signal converters to regulate the generated outputs for battery recharging. The harvested energy from the dual sources in the ambient surroundings is sufficient to drive the operation of a multi-sensing system with wireless communication capability. A maximum communication range of 1 mile is obtained between the IoT node and the gateway in both laboratory tests and actual road conditions, indicating the high efficiency and good performance of the hybrid energy harvester in practical usage scenarios.

Due to the direct and converse piezoelectric effect, piezoelectric devices can be adopted for both self-powered sensors and actuators in more diversified applications. Wang et al. (contribution 6) reported a piezoelectric microelectromechanical system (MEMS) speaker consisting of a main functional lead zirconate titanate (PZT) layer and a designed supporting layer based on rigid–flexible coupling. Compared to its rigid counterpart, the rigid–flexible coupling layer provides the speaker with a higher sound pressure level (SPL) at low frequencies, e.g., a 4.1–20.1 dB improvement for 20 Hz to 4.2 kHz. In addition, the speaker can also serve as a silent alarm in dangerous situations via the detection of oral airflows, as it is able to recognize different words based on their induced signals with unique features, and it is highly resistant to ambient interference.

The use of a cognitive radio (CR) composed of a transceiver for detecting and moving into available communication channels is a common strategy in wireless communication. Devaraj et al. (contribution 7) presented a cluster-based cooperative spectrum sensing (CBCSS) advanced algorithm with detailed investigations to improve the achievable throughput in CR. The proposed CBCSS algorithm is demonstrated for 5G and beyond-5G IoT networks, showing a better performance in achievable throughput, average cluster number and energy than the currently adopted algorithms.

In addition to the use of energy harvesters for sustainable output generation, power management circuits from energy generation to storage are also indispensable for the realization of high-efficiency self-powered IoT systems. Zhang et al. (contribution 8) proposed the design and simulation of an LDO with an outstanding low dropout voltage (100 mV) and quiescent current (nA level). To ensure high reliability and transient response, adaptive power transistors and an adaptive bias are included in the design, and the simulation results show good performance and broad adaptability for energy harvesting systems. Then, Wu et al. (contribution 9) presented a boost converter circuit with only one single inductor for managing the outputs from thermoelectric energy harvesters. A two-stage startup circuit with a three-phase operation is adopted to obtain self-startup with the single inductor. A maximum power point tracking (MPPT) strategy with coarse and fine tuning is developed to achieve the most optimal energy extraction. The boost converter is then simulated in a 180 nm CMOS process, and the results indicate that it can achieve peak efficiency and a maximum power of 88% and 3.78 mW, respectively, after self-starting at an input voltage of 128 mV.

For those who are seeking a broader overview and a more general understanding of the power management strategies in energy harvesting, the review article by Lian et al. (contribution 10) could be a good reference. The authors discuss the effective methods used to extract energy from different energy harvesters, such as photovoltaic cells, thermoelectric generators, piezoelectric generators and RF energy harvesters. Based on the distinct characteristics of each type of energy harvester, actual implementation considerations and applicable circuit designs are systematically summarized, together with their pros and cons. In particular, MPPT as an effective strategy for system efficiency improvement is highlighted and compared with other circuits in detail, echoing the work conducted by Wu et al. Overall, the two promising directions for micro-energy harvesting are further improving management efficiency via low-loss accurate control and reducing the circuit size using capacitor-based solutions.

## 3. Conclusions

The articles published in this Special Issue present important advancements in energy harvesting and self-powered sensing technologies, and the article compilation contributes to broadening the domain knowledge in the field. Although numerous exciting achievements have been made, there are still some important aspects that need to be taken into account when looking at future directions. First, in addition to the further performance improvement of energy harvesting devices, their adaptability is also essential, as the implementation environment can be complicated and unpredictable. For example, devices designed with a single transducing mechanism may suffer from the fluctuation and intermittentness of the energy source, resulting in insufficient output generation and functionality failure. One feasible approach to enhance adaptability and mitigate this issue is a hybrid generator design that combines two or more transducing mechanisms for harvesting energy from multiple sources [11]. Second, the robustness and long-term reliability of devices should be considered in practical usage scenarios in order to enable their survival when applied in extreme environments, such as high-temperature, high-pressure and underwater environments. Careful material selection, structure design and proper encapsulation should be performed prior to practical deployment [12]. Last but not least, improving the intelligence of sensors and systems is necessary and a key enabler for various smart applications [13,14,15]. The thriving of artificial intelligence (AI) technology offers a good opportunity to integrate machine learning methodologies with IoT sensors and systems. The fusion of IoT and AI technology leads to the emergence and flourishing of artificial intelligence of things (AIoT), which will no doubt conversely push the development of IoT forward.

As a final note, we hope that the articles showcased in this Special Issue are interesting and inspiring for readers who want to learn about the recent progress and conduct new research in the field. With further advancements addressing the above aspects, we also hope to see the commercialization of more diverse energy-harvesting devices and self-powered IoT nodes in the near future. The widespread use of such devices will help further promote the large-scale deployment of IoT and AIoT systems and networks, ensuring the overall welfare of human beings and the digital transformation of industries and our society.
